# A Perspective on Implementation Outcomes and Strategies to Promote the Uptake of COVID-19 Vaccines

**DOI:** 10.3389/frhs.2022.897227

**Published:** 2022-05-20

**Authors:** Meagan Pilar, A. Rani Elwy, Larissa Lushniak, Grace Huang, Gabriella M. McLoughlin, Cole Hooley, Nisha Nadesan-Reddy, Brittney Sandler, Mosa Moshabela, Olakunle Alonge, Elvin Geng, Enola Proctor

**Affiliations:** ^1^Department of Infectious Diseases, Washington University School of Medicine, Washington University in St. Louis, St. Louis, MO, United States; ^2^Department of Psychiatry and Human Behavior, Warren Alpert Medical School, Brown University, Providence, RI, United States; ^3^Center for Healthcare Organization and Implementation Research, VA Bedford Healthcare System, Bedford, MA, United States; ^4^College of Public Health, Temple University, Philadelphia, PA, United States; ^5^Implementation Science Center for Cancer Control and Prevention Research Center, Brown School, Washington University in St. Louis, St. Louis, MO, United States; ^6^School of Social Work, Brigham Young University, Provo, UT, United States; ^7^School of Nursing and Public Health, University of KwaZulu-Natal, Durban, South Africa; ^8^Bernard Becker Medical Library, Washington University in St. Louis School of Medicine, St. Louis, MO, United States; ^9^Department of International Health, Johns Hopkins Bloomberg School of Public Health, Baltimore, MD, United States; ^10^Shanti K. Khinduka Distinguished Professor Emerita, Brown School, Washington University in St. Louis, St. Louis, MO, United States

**Keywords:** implementation science, COVID-19, vaccine, implementation outcomes, implementation strategies

## Abstract

Recent articles have highlighted the importance of incorporating implementation science concepts into pandemic-related research. However, limited research has been documented to date regarding implementation outcomes that may be unique to COVID-19 vaccinations and how to utilize implementation strategies to address vaccine program-related implementation challenges. To address these gaps, we formed a global COVID-19 implementation workgroup of implementation scientists who met weekly for over a year to review the available literature and learn about ongoing research during the pandemic. We developed a hierarchy to prioritize the applicability of “lessons learned” from the vaccination-related implementation literature. We identified applications of existing implementation outcomes as well as identified additional implementation outcomes. We also mapped implementation strategies to those outcomes. Our efforts provide rationale for the utility of using implementation outcomes in pandemic-related research. Furthermore, we identified three additional implementation outcomes: availability, health equity, and scale-up. Results include a list of COVID-19 relevant implementation strategies mapped to the implementation outcomes.

## Introduction

The coronavirus disease 2019 (COVID-19) pandemic vividly exemplifies an implementation crisis: life-saving remedies exist, but their adoption and spread have lagged world-wide. Many longed for vaccines and heralded their development. However, many medical and public health experts were surprised at the skepticism, hesitancy, and outright resistance to available vaccines, even as the pandemic led to sky-rocketing rates of mortality and morbidity and compromised even the most resource-rich health systems. Despite recommendations from the World Health Organization, we are still far from achieving the proposed goal of 70% vaccination coverage globally (1). Though there is some debate as to whether 70% vaccination is still sufficient due to the emergence of new variants (2), the challenges with vaccine program implementation remain.

Implementation science is well-suited to tackle crises like the COVID-19 pandemic. But what specifically does it offer? This Perspective article addresses one part of this question by demonstrating the importance of conceptualizing vaccine roll-out through the lens of two implementation science concepts: implementation outcomes and implementation strategies. These concepts help clarify some of the most pressing questions about the implementation challenges surrounding vaccination programs: What needs to be achieved, and how do we get there? This article is not meant to be an exhaustive review of current research; rather, it is intended to provide the reader with guidance on how we might more clearly conceptualize the implementation outcomes—both anticipated and actual (3)—that are most relevant for increasing the uptake of COVID-19 vaccines. Additionally, we will provide examples of implementation strategies, developed to ensure successful implementation outcomes, to better aid researchers and practitioners in evaluating the implementation of COVID-19 vaccines.

## Applying an Implementation Science Lens to the Covid-19 Pandemic

Implementation science is the “study of methods to promote the systematic uptake of research findings and other evidence-based practices into routine practice” (4). This field seeks to provide guidance in cases where evidence-based interventions exist but are poorly implemented or, in some instances, not implemented at all. In the case of the COVID-19 pandemic, an intervention—a vaccine—exists, but it has been underutilized for myriad reasons (e.g., issues surrounding supply and distribution, mistrust, misinformation, vaccine hesitancy). Implementation science provides an opportunity to apply existing methods to study these challenges and improve the uptake of the COVID-19 vaccine.

Implementation science is inherently pragmatic and involves real-world, diverse populations, data collection that is meaningful and actionable, and a focus on the application of an evidence-based practice (e.g., vaccination) in local contexts (5). Thus, ensuring the successful uptake of COVID-19 vaccines globally requires a pragmatic, low burden assessment of stakeholders' perceptions of implementation outcomes related to vaccination (e.g., acceptability, cost) (6), as well as specific and operationalized implementation strategies to address barriers to vaccine uptake (7).

Previous articles have underscored the importance of incorporating implementation science concepts into pandemic-related research (8–10). For example, it is vital to engage stakeholders from project inception and consider context during implementation, as factors such as available resources, policy support, health system and population characteristics can impact the uptake of an intervention (9). Additionally, implementation science theories and methods can help inform the equitable development, implementation, and evaluation of interventions to address health disparities and promote health equity (11–14). However, there has been a limited research focus to date regarding implementation outcomes that may be unique to COVID-19 vaccinations and how to utilize implementation strategies to address vaccine program-related implementation challenges. To address these gaps, we formed a global COVID-19 implementation workgroup of implementation scientists who met weekly for over a year, to review the available literature and learn about ongoing research during the pandemic. Our efforts resulted in a list of implementation outcomes that can be used to evaluate the implementation of vaccine programs globally. Likewise, we have compiled a list of implementation strategies, which can be mapped onto the aforementioned outcomes to address common challenges related to vaccine program implementation.

Given this novel disease and the unprecedented times, evidence directly related to COVID-19 is still developing. As a result, our recommendation for implementation strategies have been informed by COVID-19 evidence, as well as previous public health efforts, which we believe are transferable or applicable to this pandemic. To aid in this conceptualization of evidence, our team constructed the following hierarchy by which to prioritize recommendations and “lessons learned.” We drew from existing evidence hierarchies [e.g., GRADE (15) and AGREE II (16)] and used a consensus-based approach among the authors similar to that espoused for developing guideline recommendations [e.g., the DECIDE framework (17–19)]. The implementation science base of evidence to guide strategy selection for COVID-19 mitigation ranges from scant to very strong.

1. Direct evidence from COVID-19 vaccination: scant and emergent, especially early in the pandemic.
2. Evidence from other preventative strategies used during COVID-19 (e.g., masking, social distancing): emergent.
3. Evidence from vaccinations of non-COVID-19 diseases that are respiratory, novel, or heterogeneous in severity, or during a pandemic (e.g., influenza): solid to strong.
4. Evidence from vaccination for other infections (e.g., measles, polio): strong.
5. Evidence from other health conditions: very strong.

## Implementation Outcomes

Implementation outcomes provide a means to evaluate the implementation success of interventions, treatments, policies, and protocols and are distinct from other, traditionally measured outcomes, such as service system and clinical outcomes (6). In the case of COVID-19 prevention and treatment, service system outcomes include the timeliness and efficiency of the health system, and perceived equity and patient-centeredness of treatments, while clinical outcomes reflect population and patient health and safety, as well as satisfaction with treatment options, including vaccines.

In 2011, Proctor and colleagues developed the Implementation Outcomes Framework as a way to conceptualize and measure eight distinct implementation outcomes—acceptability, adoption, appropriateness, cost, feasibility, fidelity, penetration, and sustainability (6). This framework has since been widely cited and applied within the implementation science community. However, based on our review of the literature, there are additional outcomes that could—and should—be considered within the context of the COVID-19 pandemic to provide a more comprehensive evaluation of implemenation of vaccine programs. As a result, we have operationalized the eight well-known implementation outcomes based on their relevance to the COVID-19 pandemic and have proposed three additional outcomes (availability, health equity, and scale-up) for consideration (Table 1).

**Table 1:** Operationalization and addition of implementation outcomes unique to COVID-19.

**Implementation outcome**	**Definition**	**Application to COVID-19**
Acceptability^*^	The perception among stakeholders that a given treatment, service, practice, or innovation is agreeable, palatable, or satisfactory	Vaccine implementation requires a minimum level of acceptability among policy makers, healthcare providers, community leaders, parents/caregivers, and those eligible for the vaccine. Vaccine acceptability is undermined by misinformation, lack of trust in governments and health systems, and anti-vaccine beliefs and attitudes.
Adoption^*^	The intention, initial decision, or action to try or employ a new treatment or innovation—also referred to as “uptake”	Vaccine adoption can be impacted by external mandates, vaccine supply, organizational culture and climate, and healthcare providers' willingness to recommend and administer the vaccine.
Appropriateness^*^	The perceived fit, relevance, or compatibility of the treatment or innovation for a given practice setting, provider, or consumer; and/or the perceived fit of the innovation to address a particular issue or problem	The appropriateness of vaccines is influenced by organizational- and individual-level factors, such as existing resources within an organization, healthcare providers' knowledge and perceptions of the vaccine, and patients' ability and willingness to receive the vaccine.
Availability^**^	The supply of a new treatment or innovation at any one time	Vaccines can be utilized only when they are available, and the sufficiency of their availability depends on supply, the size of population to be covered, and the size of priority groups receiving vaccines.
Cost^*^	The cost impact of an implementation effort	The cost of vaccine implementation includes multiple levels, such as the cost to procure vaccine doses (e.g., national financing), administer doses (e.g., organizations' costs to pay employees, restructure existing processes, etc.), incentivize doses (e.g., organizations paying employees to be vaccinated), and receive doses (e.g., individuals' costs to travel, take time off work, etc.).
Feasibility^*^	The extent to which a new treatment or innovation can be successfully used or carried out in a given setting	The feasibility of vaccine implementation is affected by supply, as well as geographic factors, transportation and storage structures, and the capabilities, time, and training of vaccine providers.
Fidelity^*^	The degree to which a treatment or innovation was implemented as it was prescribed in the original protocol or as was intended by program developer	Vaccines must be stored, distributed, and delivered with strict fidelity to dosing and safety requirements.
Health Equity^**^	Fair access to a treatment or innovation without avoidable or remediable differences among groups of people	Vaccine equity requires consideration of sub-groups, including groups with priority needs and those that differ by social, economic, demographic, and geographical factors linked to systemic disadvantage.
Penetration^*^	The integration of a treatment or innovation within a service setting and its subsystem	The penetration of a vaccine program is impacted by myriad factors, including policies, organizational culture, personal beliefs, and the supply of the vaccine itself.
Scale-up^**^	Deliberate efforts to increase the impact of successfully tested health innovations so as to benefit more people and to foster policy and program development on a lasting basis (20)	The potential for scale-up is affected by factors such as vaccine hesitancy, supply shortages, financial and technological resources, political will, and existing infrastructure (e.g., cold-chain system infrastructure and ample workforce).
Sustainability^*^	The extent to which a treatment or innovation is maintained or institutionalized within a service setting's ongoing, stable operation	The sustainability of vaccination efforts and programs must be incorporated into existing both national and organizational structures (e.g., allocating financial and human resources), workflows (e.g., restructuring employee roles and responsibilities), and/or programs (e.g., bundling with existing programs).

* *Definitions are adapted from the Proctor et al. 2011 paper (6)*.

** *Team added constructs—availability (21–24), health equity (25–27), and scale-up (28)—based on theoretical and practical application*.

Evaluations of COVID-19 mitigation efforts could have been—and still have potential to be—more precise and robust with a greater focus on implementation outcomes. These outcomes can play three important roles in relation to COVID-19 control. First, they allow researchers and implementers to focus efforts on assessing the baseline or starting point, thereby quantifying the gap between desired and achieved outcomes. Vaccines need to “have an efficacy of at least 70% to prevent an epidemic and of at least 80% to largely extinguish an epidemic without any other measures (e.g., social distancing)” (29). Within the context of COVID-19 vaccination, where many countries included other mitigation measures in their efforts to control the pandemic, such as mask wearing and social distancing, this could include monitoring the vaccination status of a population. In addition to quantifying gaps in implementation, incorporating implementation outcomes into vaccine program planning and evaluation also provides a source of accountability for implementers at the national-, state-, or local-levels. The additional implementation outcomes of availability and health equity are essential for increasing vaccine uptake. Vaccines can be utilized only when they are available, and the sufficiency of their availability depends on supply, the size of population to be covered, and the size of priority groups receiving vaccines. Similarly, vaccine equity requires consideration of sub-groups, including groups with priority needs for vaccination and those that differ by social, economic, demographic, and geographical factors linked to systemic disadvantage.

Second, implementation outcomes provide a direction for implementation efforts. For instance, factors such as the feasibility and sustainability of public health programs may be prioritized when implementation outcomes are incorporated into the planning and evaluation process. These outcomes can also highlight potential barriers or facilitators that may arise during implementation and help researchers identify implementation strategies to address potential challenges. As we have seen over the past 2 years, myriad challenges can arise during vaccine development, distribution, and implementation. For example, factors such as unfamiliar technology or a lack of staff buy-in may decrease the feasibility of implementing a COVID-19 vaccine program. The additional implementation outcome of scale-up we have defined illustrates how vaccine scale-up is affected by factors such as vaccine hesitancy, supply shortages, financial and technological resources, and existing infrastructure (e.g., cold-chain system infrastructure and ample workforce). Scale-up differs from the original penetration implementation outcome, which refers to the degree to which an intervention has infiltrated a service system. Scale-up, on the hand, is broader, encompassing multiple service systems thereby requiring different strategies at higher socioecological levels. Additionally, the deluge of information disseminated through social media has increasingly delivered misinformation impacting acceptability of COVID-19 vaccines globally. As a result, specific strategies can be selected based on the implementation context. Challenges with technology could be addressed through the provision of technical assistance, while buy-in could be encouraged by identifying champions within the organization. Finally, vaccine-related misinformation could be counteracted through a targeted public health campaign emphasizing the transparency of the vaccine development process, and using jargon-free messaging that takes into account socioeconomic and cultural factors, as well as personal and media sources needed for communicating with specific populations (30).

Third, implementation outcomes can increase the precision of evaluation efforts surrounding public health programs. Too often, public health systems' data collection is limited to clinical outcomes (e.g., number of COVID-19 cases, hospitalizations, and mortality). However, measuring intermediate outcomes, such as acceptabilty, adoption and fidelity, paints a more complete picture of program implementation and illuminates the challenges to current implementation efforts. For example, several global surveys conducted throughout the pandemic have assessed the degrees of acceptability of COVID-19 vaccines over time (31, 32). Important findings from these surveys have revealed that those who trust their governments and their messaging (31) and receive information and guidance from healthcare providers (32) reported that they will be more likely to become vaccinated against COVID-19. In another project, COVID-19 vaccine acceptability among patients and employees of a large integrated healthcare system in the United States was assessed through surveys (33) and interviews (34). Specific, tailored communication strategies were then created to overcome identified barriers to receiving a vaccine and were disseminated widely across the healthcare system, for use in one-to-one conversations between trusted providers and patients, and among employees (34). The additional information regarding, for example, program acceptability can enable implementers to better evaluate program efforts and adapt practices to promote the uptake of COVID-19 vaccines.

## Implementation Strategies

Implementation strategies are defined as “methods or techniques used to enhance the adoption, implementation, and sustainability of a clinical program or practice” (7). Implementation scientists have emphasized the importance of identifying and compiling evidence-based strategies (35), selecting strategies based on implementation context and barriers (36), and specifying the use of implementation strategies in research (7). Building on this body of work, our team set out to map implementation strategies onto specific implementation outcomes and challenges to provide guidance for researchers and practitioners to increase the uptake of vaccines and aid in the scale-up and spread of successful vaccination programs.

After identifying and conceptualizing critical implementation outcomes to assess to determine the effectiveness of COVID-19 vaccine implementation globally (Table 1), our research group considered some of the most common challenges encountered during vaccine program implementation (e.g., mistrust, misinformation, vaccine hesitancy, supply issues). We then compiled a range of implementation strategies to determine which of these may be most useful in overcoming common implementation challenges and increasing uptake of COVID-19 vaccines. We present the implementation outcomes, implementation challenges, and the implementation strategies our team identified, and details on these strategies (e.g., specified actions or tools) identified in the literature to improve implementation success (Table 2).

**Table 2:** Implementation outcomes, challenges, and strategies applicable to the COVID-19 vaccine program implementation.

**Implementation outcome**	**Implementation challenge**	**Implementation strategy**	**Example**
Acceptability	Vaccine hesitancy (broad)	Plan: Identify hesitancy in the population or subgroups and tailor intervention efforts to reach them	Used the Guide to Tailoring Immunization Programs (37, 38) to identify subgroups with low immunization rates, diagnose factors impacting vaccine hesitancy, and tailor programs to addresses the factors leading to low vaccine acceptance in the subgroup (39) Used the Social Mobilization Network (SMNet) to target resistance at multiple levels through effective, personalized health communication (40)
	Low vaccine demand	Educate: Receive recommendation from a trusted source	Promoted demand for vaccines through a personalized recommendation from a trusted healthcare provider in communities of color (41)
	Limited knowledge or awareness of vaccines	Plan: Design and implement a health campaign	Created a vaccination program through the Cameroon Baptist Convention Health Services that targeted schools, clinics, churches, and regarding HPV and cervical cancer (42) Implemented a national vaccination campaign through school and community outreach sessions (43) Used nation-wide campaign, National Immunization Days (NIDs), to administer vaccines in locations across the country (44)
Adoption	Low levels of vaccine awareness	Educate: Train health workers/community volunteers in information, education and communication tactics	Harnessed the power of social networks and trained community volunteers to and increase awareness of and support for HIV vaccine research in minority populations (45)
	Misconceptions about the vaccine and its effects	Plan/Educate: Use health communication strategies to address mistrust	Recommendations to create positive vaccine narratives and use positive emotional appeals (e.g., hope and job in receiving a vaccine) to counteract negative emotions (e.g., fear, anger, mistrust) surrounding vaccination (46) Conducted a series of town hall meetings to address concerns and misinformation raised by healthcare workers and staff (47)
	Vaccine hesitancy (mistrust in science or the vaccine)	Plan/Educate: Create health communication materials	Created a digital infographic to promote trust in science, reduce the believability of misinformed narratives, and increase the likelihood of engaging in preventive behaviors (48)
Appropriateness	Low vaccine demand and mistrust in the community	Plan: Tailor outreach efforts and communication strategies to subgroups (e.g., race/ethnicity, gender, rural areas)	Recommendations regarding outreach—Efforts should be led by physicians reflecting the diversity of the subgroups (e.g., Black physicians affiliated with historically Black medical institutions to target communities of color; physicians from well-respected medical institutions in Republican-leaning states to target conservative states) (49)
	Vaccine hesitancy (broad)	Plan: Create persuasive public health communication plans tailored to an individual's level of vaccine hesitancy	Segmented portions of the population and target health communication efforts to their identified barriers (50) Utilized vaccine messaging that address the personal benefits of vaccination (e.g., prevention of chronic illness) to target the hesitant population (51)
	Vaccine hesitancy (broad)	Plan: Use effective mass communication strategies	Recommendations to emphasize transparency regarding vaccine-related health communications (e.g., safety, efficacy, vaccine development, distribution, and cost) (52)
	Vaccine hesitancy (mistrust in science or the vaccine)	Educate: Provide training to promote cultural competence	Recommendations to train and equip healthcare providers, particularly when working with historically marginalized groups (53)
Availability	Limited number of suppliers	Finance; Alter incentive structure by developing advance purchase commitments	Need an integrated policy approach that preserves incentives for market entry and innovation in the vaccine industry while addressing vaccine concerns and increasing immunization funding and reimbursement for both providers and patients (54)
	High research and development and production costs	Finance: Access new funding through government subsidies for basic vaccine research	Increased funding through the US Biomedical Research and Development Authority, resulting in over $19.3 billion to facilitate COVID-19 vaccine development (55)
	Safety problems leading to increased regulatory requirements.	Policy context: Change liability laws to provide protection for manufacturers	Established a COVID-19 vaccine injury no-fault compensation scheme in South Africa to facilitate COVID-19 vaccine rollout (56)
	Storage availability	Policy context: Identify barriers and facilitators and test new workflows	Increased the frequency of transport capacity to reduce storage bottlenecks and increase vaccine availability (57)
Cost	Limited economic resources	Finance: Engage and mobilize stakeholders and payers	Recommendations to ensure adequate operational funds are mobilized in readiness for the vaccination exercise based on the country micro-plans (58)
	Limited economic resources	Finance: Include COVID-19 vaccine strategy government budgets	Recommendations to estimate funding needs and align cost plans with existing resources while minimizing fragmentation for existing programs (58)
	Limited economic resources	Finance: Provide financial incentives (in settings with low immunization coverage)	Utilized trusted vaccine “ambassadors,” SMS reminders, and low-cost incentives (i.e., mobile phone credit) to increase vaccine uptake (59)
	Limited economic resources	Finance: Identify potential new sources of revenue	Recommendations to facilitate dialogue and alignment with the budget and planning departments of the Ministry of Health, Ministry of Finance, and the funding partners (58)
Feasibility	Low vaccine demand	Restructure: Bundle vaccine efforts with existing community programs	Recommendations regarding how vaccination programs could be offered alongside existing services and valued community initiatives, such as nutritional support and food supplementation programs (60) Recommendations to consider other factors when bundling programs: similarities in target groups, logistical requirements, skill levels required for healthcare staff (61)
	Geographic inaccessibility	Restructure: Decrease geographic barriers to vaccine uptake	Included community organizations, such as schools, as centers for vaccine campaign administration (43) Employed strategies such as door-to-door visits to spread awareness of vaccine program goals and local vaccination sites (60)
Fidelity	Compliance with public health recommendations	Policy context: Develop enforcement policies regarding vaccination	Recommendations to consider factors such as control aversion, trust in the government, and the degree of enforcement when designing enforcement vs. voluntary policies (62) Recommendations for policymakers to develop programs that optimize identification and treatment of those with disease while minimizing the use of invasive measures, such as involntary detention of noncompliant patients or forced administration of vaccinations (63)
Fidelity	Inability to track disease spread and report cases	Quality management: Strengthen surveillance systems and establish robust system for capturing and tracking cases Examples: Provide supervision; use desk and field reviews to assess quality of AFP surveillance	Recommendations to improve surveillance system's functioning, sensitivity, and quality despite challenges such as a large national geographic expanse, zones with chronic insecurity and inaccessibility, and a lack of capacity and infrastructure (64)
Health equity	Structural racism	Educate: Provide equity training for implementers	Recommendations to provide training, education, or opportunities for reflection in health equity, addressing structural racism, and/or promoting antiracism approaches with respect to our research, institutions, and community partnerships (e.g., Public Health Critical Race Praxis) (65)
	Unequal power dynamics with stakeholders	Plan: Include early and ongoing engagement with stakeholders in both decision-making and prioritization	Recommendations to promote transparency, consideration of power dynamics, equitable sharing of resources, respect of community values, and inclusion of racially/ethnically diverse partners as equitable decision-makers early and often (65)
	Vaccine hesitancy (broad)	Plan: Engage community partners to promote vaccine-related information-sharing and build trust with marginalized communities	Increased vaccine acceptance by waiting for safety data to be more robust, knowing more about the vaccine, and receiving a recommendation to take the vaccine from a trusted healthcare provider (41) Engaged youth group members and significantly enhanced the ability of vaccination teams to vaccinate chronically missed children (66) Engaged pastors as trusted messengers; created partnerships with shared responsibility and power; and co-created solutions with faith leaders and their community, governments and institutions (67) Utilized local community members to spread information about vaccination events, which was more effective than mass media advertisements (68)
	Low vaccine demand Vaccine hesitancy (mistrust in science or the vaccine)	Plan: Create micro-plans with hard-to-reach communities at center of plans	Elicited immunization preferences for six program characteristics (e.g., location, use of incentives, bundling with existing services) to create a targeted approach for implementation (60) Utilized local and religious leaders to enhance community knowledge of vaccination campaigns (43)
	Low vaccine demand	Plan: Utilize social networks to increase reach and uptake	Used social network methods to identify and recruit to provide access to high-risk youth who may be critical recipients of a future vaccine (38)
Penetration	Low vaccine demand	Restructure: Enable community health workers to promote vaccine uptake	Utilized existing community structures such as churches to spread preventive care messages and facilitate vaccine promotion (69)
	Low vaccine demand	Quality management: Use patient-held web-based portals and computerized reminders increase immunization coverage rates	Used text messaging, immunization campaign websites, patient-held web-based portals, and computerized reminders and standing orders for physicians to increase immunization coverage rates (70)
	Limited advocacy for an implementation	Plan/Quality management: Identify and engage policy entrepreneurs and champions in various levels of government, user organizations, and the broader community	Recruited individuals who were highly motivated to move forward with innovations and advocate for their promotion and adaptation at organizational or bureaucratic levels (20)
Scale-up	Lack of dynamic partnerships	Quality management: Assess the strengths and weaknesses of the user organization (e.g., public sector health service system, NGO, etc.) and develop strategies to build capacity	Recommendations to identify how user organizations' resources, staffing, organizational culture, and leadership structures will affect program scale-up (20)
	Limited organizational capacity to implement	Plan/Educate: Ensure the team has necessary skills and capacities to implement a vaccine program	Recommendations to conduct program evaluation, management, training, economic evaluation, fundraising, health communication, and writing while emphasizing the importance of cultural knowledge (20)
	Limited consideration of external influences when developing implementation program goals	Quality management/Restructure: Identify the environmental factors influencing scaling up and understand how they affect the process	Recommendations to consider how policy/politics, bureaucracy, and socio-economic/cultural contexts will directly impact vaccination program scale-up prior to implementation (20)
Sustainability	Vaccine hesitancy (broad)	Plan/Educate: Prepare materials for healthcare workers to better integrate into routine practice	Recommendations to (1) prepare a list of common vaccine questions; (2) develop a list of effective responses; and (3) train and practice with staff to response to patients' concerns (50)
	Limited organizational capacity to implement	Restructure: Bundle vaccine efforts with existing community programs	Recommendations to consider multi-level factors when bundling programs: similarities in the availability of funding, logistical requirements, political support, and level of burden (i.e., to ensure that bundling does not disrupt service delivery) (61)
	Low vaccine demand Vaccine hesitancy (mistrust in science or the vaccine)	Plan: Build buy-in with stakeholders	Engaged trusted community figures, such as community influencers, local religious leaders, healthcare providers, and parents (41, 49, 52, 71)

In many cases, we found that several implementation strategies were needed to address these complex, multi-level implementation challenges (5), especially prevalent in vaccination programs. For example, Rajkumari and colleagues identified two barriers related to the uptake of a measles-rubella vaccine in India—limited knowledge of the vaccine and geographic inaccessibility—which impacted the vaccine program's acceptability and feasibility, respectively (43). As a result, the team selected implementation strategies to address these barriers, which included creating a national health campaign and administering vaccines through community organizations, such as schools (43). Momplaisir et al., explored attitudes and beliefs related to COVID-19 vaccinations within Black communities in the United States (41). Though participants reported low demand and high levels of vaccine hesitancy, they also identified that recommendations from a healthcare provider might increase their trust in the vaccine's safety and efficacy (41). These findings illustrate the importance of engaging community partners and promoting trust, particularly in historically underserved communities, to increase the acceptability and equitable distribution of the COVID-19 vaccine.

## Discussion

The COVID-19 pandemic is an evolving situation, and implementation science needs to respond accordingly. Many of our theories and study designs have an implicit long period of time that's required to assess implementation (72). However, this situation requires rapid assessments and evaluations, as well as the measurement of implementation outcomes that may have been previously overlooked (73).

Our team's activity provided evidence that the original Proctor implementation outcomes are still essential for examining the success of COVID-19 vaccination programs globally; yet three outcomes—those of availability, health equity and scale-up—are welcome additions to the implementation outcomes framework. Addressing implementation challenges related to availability, for example, allows governments and policymakers to focus on the earlier, or pre-implementation, factors that support widespread vaccine scale-up, such as increasing vaccine suppliers and providing incentives and additional funding structures to address high research and development costs. In certain contexts, policies may also need to change regarding manufacturing liability, which is essential for increasing the availability and eventual scale-up of vaccines. Vaccine availability is also impacted by storage issues and can be mitigated by additional infrastructure support, such as increasing transportation, which is needed for future scale-up.

Health equity, a critical part of implementation science, and fair access to COVID-19 vaccines has been a continual challenge globally. Health equity is also closely related to the implementation outcomes of availability and scale-up. For example, when there is limited vaccine supply or organizational capacity for vaccine program implementation, power struggles that exist among stakeholders with varying social, economic, demographic, and geographical differences may lead to an inequitable distribution of vaccines (e.g., vaccines only being available to groups whose power aligns with the organizations distributing vaccines). Additionally, greater distrust in science and vaccines within historically underserved populations may further impede vaccine acceptance and uptake. This underscores the importance of considering the myriad external influences that lead to misinformation campaigns and distrust when assessing the potential for vaccine program scale-up.

Through engagement of a collaborative working group on global vaccine implementation, our team was able to apply a well-known implementation science framework to current and past literature, policies, and country-level knowledge about vaccines. As a result, we identified three additional implementation outcomes and compiled a list of specific implementation strategies that can be applied at multiple levels to increase vaccine uptake.

## Data Availability Statement

The original contributions presented in the study are included in the article/supplementary material, further inquiries can be directed to the corresponding author/s.

## Author Contributions

EP, EG, MM, and OA contributed to conception of the study. MP, AE, and EP wrote the first draft of the manuscript. GH and LL wrote portions of the manuscript. All authors contributed to manuscript revision, read, and approved the submitted version.

## Funding

This work was supported in part by the National Institute of Allergy and Infectious Diseases (No. K24AI134413). EP effort was supported by the Implementation Science-Entrepreneurship Unit of the Washington University Institute of Clinical and Translational Sciences (No. UL1TR002345) from the National Center for Advancing Translational Sciences of the National Institutes of Health; the National Institute of Mental Health (No. R25MH080916); National Cancer Institute (No. P50CA244431); National Institute of Mental Health (No. 5P50MH113662); Patient-Centered Outcomes Research Institute Award (No. TRD-1511-33321).

## Author Disclaimer

The findings and conclusions in this paper are those of the authors and do not necessarily represent the official positions of the National Institutes of Health. Additionally, the views expressed in this article are those of the authors and do not necessarily reflect the position or policy of the Department of Veterans Affairs or the United States government.

## Conflict of Interest

The authors declare that the research was conducted in the absence of any commercial or financial relationships that could be construed as a potential conflict of interest.

## Publisher's Note

All claims expressed in this article are solely those of the authors and do not necessarily represent those of their affiliated organizations, or those of the publisher, the editors and the reviewers. Any product that may be evaluated in this article, or claim that may be made by its manufacturer, is not guaranteed or endorsed by the publisher.
